# How community-dwelling seniors with multimorbidity conceive the concept of mental health and factors that may influence it: A phenomenographic study

**DOI:** 10.3402/qhw.v7i0.19716

**Published:** 2012-12-13

**Authors:** Åke Grundberg, Britt Ebbeskog, Madeleine Abrandt Dahlgren, Dorota Religa

**Affiliations:** 1 Department of Neurobiology, Care Sciences and Society, Alzheimer Disease Research Centre (KI-ADRC), Karolinska Institutet, Stockholm, Sweden; 2 Division of Nursing, Department of Neurobiology, Care Sciences and Society, Karolinska Institutet, Stockholm, Sweden; 3 Deptartment of Medicine and Health, Faculty of Health Sciences and Department of Behavioural Sciences and Learning, Linköping University, Linköping, Sweden

**Keywords:** Aged, care of older people, mental health promotion, municipal care, nursing, phenomenography, primary health care

## Abstract

Multimorbidity, that is, the coexistence of chronic diseases, is associated with mental health issues among elderly people. In Sweden, seniors with multimorbidity often live at home and receive care from nursing aides and district nurses. The aim of this study was to describe the variation in how community-dwelling seniors with multimorbidity perceive the concept of mental health and what may influence it. Thirteen semi-structured interviews were analysed using a phenomenographic approach. Six qualitatively different ways of understanding the concept of mental health and factors that may influence it, reflecting key variations of meaning, were identified. The discerned categories were: mental health is dependent on desirable feelings and social contacts, mental health is dependent on undesirable feelings and social isolation, mental health is dependent on power of the mind and ability to control thoughts, mental health is dependent on powerlessness of the mind and inability to control thoughts, mental health is dependent on active behaviour and a healthy lifestyle, and mental health is dependent on passive behaviour and physical inactivity. According to the respondents’ view, the concept of mental health can be defined as how an individual feels, thinks, and acts and also includes a positive as well as a negative aspect. Social contacts, physical activity, and optimism may improve mental health while social isolation, ageing, and chronic pain may worsen it. Findings highlight the importance of individually definitions of mental health and that community-dwelling seniors with multimorbidity may describe how multiple chronic conditions can affect their life situation. It is essential to organize the health care system to provide individual health promotion dialogues, and future research should address the prerequisites for conducting mental health promotion dialogues.

More people are reaching an advanced age, many of whom are frail and suffer ill health due to multiple diseases and various acute illnesses. The prevalence of disease and impairment increases with advancing age, as do limitations on the ability to manage the activities of daily living. The concept of multimorbidity is often used when researchers and public health institutions describe frail elderly people with multiple chronic diseases having comprehensive and complex need of care and support. Multimorbidity, that is, the coexistence of chronic diseases, results in frequent general practice appointments, long hospital stays, and higher health care costs. Multimorbidity by association with mental health issues leads to poor health-related quality of life (HRQOL), suicide, and high costs to society.

In epidemiology, multimorbidity has been defined as the co-occurrence of two or more chronic diseases in one person (Van den Akker, Butinx, Metsemakers, Roos, & Knottnerus, 1998). Multimorbidity is common in several Western countries, both in the general population and among primary care patients (Fortin et al., 2004). For instance, in general practice settings in the Netherlands, the prevalence of multimorbidity increases with higher age to 78% among persons aged 80 years and over (Van den Akker et al., 1998). Multimorbidity is also associated with psychological distress (Fortin et al., 2006), generally poor quality of life (Fortin, Lapointe, Hudon, Ntetu, & Maltais, 2004), and depressive disorders (McEvoy & Barnes, 2007; Spangenberg, Forkmann, Brähler, & Glaesmer, 2011). Individuals with mental health problems are more likely to seek help from primary care rather than mental care providers (Luoma, Martin, & Pearson, 2002). Late-life depression is common in primary care (Lyness, Caine, King, Cox, & Yoediono, 1999), and depression is frequently treated. Despite the fact that depression is a socially and physically disabling condition (Unützer et al., 2000) and is the principal risk factor for suicide in later life (Conwell, Duberstein, & Caine, 2002), there are still barriers to its effective treatment (Ford, 2000). Patient's barriers include poor adherence to antidepressant medication and the stigma associated with mental illness (McEvoy & Barnes, 2007).

The organizations that provide care and services to old people with complex needs vary between countries, depending on the structure of the health care system and its financial situation (Ryan, McCann, & McKenna, 2009). The definition of primary care varies between countries. Swedish primary care has developed from a philosophic viewpoint based on quality, continuity, accessibility, co-operation, and a holistic view (Strandberg, Ovhed, Borgquist, & Wilhelmsson, 2007). In Sweden, health-promoting activities are considered important, especially in primary care, where general practitioners and registered specialist nurses known as district nurses have the main responsibility for health promotion activities. Their responsibilities are stipulated by law and include health promotion among patients of all ages (Wilhelmsson & Lindberg, 2009).

Western countries are currently experiencing a continuous increase in informal care service provision to seniors in their homes (Klein, 2008). In Sweden, seniors with multimorbidity who live in their own home often need significant care from the public sector. Irrespective of the international differences between health care providers, the increase in seniors’ need for health care and support is common to several Western countries. Swedish government reforms have led to the provision of more care and service in ordinary housing by nursing aides or enrolled nurses (National Board of Health and Welfare, 1999). Seniors living in their own homes can be granted support from the municipal home help service in accordance with the Social Service Act (SFS, 2001, p. 453). The support varies according to the client's need and facilitates daily life in areas such as purchasing groceries, cleaning, and personal care.

Health can be seen as the opposite of disease, or as a continuum from health to disease, that is, the more health the less disease or illness. Another way of looking at health is to view health and illness as two co-existing independent dimensions (Medin & Alexandersson, 2000). Health must be understood in a social and cultural context related to age, sex, social and economic status, education, and so on (Olin Lauritzen, 2001). Health and mental health are concepts with scales containing different positions. Both researchers and lay persons regard health as something to strive for, but sometimes the words good health and bad health are used, indicating that negative as well as positive health is included in the concept (Brülde & Tengland, 2003). Jahoda (1953) stated that “mentally healthy perception means a process of viewing the world so that one is able to take in matters one wishes different without distorting them to fit those wishes” (p. 349). Thus, one can infer that mentally ill people often have distorted images of themselves. Taylor and Brown (1988) challenged this widely held criterion for mental health. They argued that positive illusions, which was defined “as beliefs that depart from reality” (p. 194), promote good mental health. The goal of health promotion should be to improve mental, social, and physical health, in combination with the prevention of mental, social, and physical ill health (Downie et al., 1996). Mental health promotion is defined by the World Health Organization as the creation of living conditions and environments that support mental health and allow people to adopt and maintain healthy lifestyles (WHO, 2010). Physical illness is strongly associated with mental disorders (Jacobi et al., 2004) such as depression (Smyth, 2009). An earlier review showed that between 20% and 50% of older adults with long-term physical conditions have clinically significant symptoms of depression (Djernes, 2006). Other examples of risk factors are living alone, social isolation (Skoog, 2004), and pain (Bair, Robinson, Katon, & Kroenke, 2003). According to Svedberg, Arvidsson, Svensson, and Hansson (2008), the essence of mental health promotion is empowerment together with educational and practical support provided by means of a good alliance.

Self-perception is the key to our understanding of mental health. Self-insight and accurate self-perception are viewed as the marks of a well-adjusted and healthy personality, because good mental health naturally means having an accurate view of reality (Gana, Alaphilippe, & Bailly, 2004). Marton and Booth (1997) argued that to make sense of how people handle a situation, we have to understand the different ways in which they experience it. As an increasing number of international studies show that mental health issues may occur among elderly with long-term conditions, it is important to know how they perceive the concept of mental health. It is equally needed to analyse factors influencing mental health, especially when this has not been adequately studied.

## Aim

The aim of this study was to describe variation in how community-dwelling seniors with multimorbidity perceive the concept of mental health and what may influence it.

## Methods

The study used a descriptive, qualitative design with a phenomenographic approach. This approach was chosen to describe the variation of how the respondents perceive the concept of mental health and what may influence it. The aim of traditional phenomenographic research is to investigate the different ways in which people understand a phenomenon, in this case mental health. The different ways of understanding have both “what” and “how” aspects. The “what” aspect tells us what is in the subject's focus, and the “how” aspect describes how meaning is created. In phenomenography, a distinction is made between the first-order perspective, which has to do with facts, and the second-order perspective, which concerns the individual's perception of the phenomena (Marton & Booth, 1997).

Interviews, and especially semi-structured ones, serve as the basis for data collection in this research (Marton & Booth, 1997). The phenomenographic analysis focuses on differences and similarities between individual statements that lead to defined conceptions (Marton, 1994). These different ways of perceiving, conceiving, or understanding are presented in the form of descriptive categories (Dahlgren & Fallsberg, 1991; Marton & Pong, 2005), the purpose of which is to relate the conceptions to each other and to the statements (Marton, 1994). The categories of description are the researcher's abstractions of the different ways of understanding, which have been identified. They refer to a collective level and describe the different ways the phenomenon *can* be understood. All the categories of description, the outcome space, constitute the results of a phenomenographic study. The different categories in the outcome space are usually related to one another in a hierarchical way (Marton & Booth, 1997).

### Setting and participants

The study took place in the participants’ homes in an urban area in Sweden. A purposive sampling technique was used in a deliberative and non-random fashion to achieve a certain goal (Dahlgren, Emmelin, & Winkvist, 2007). We wanted to identify elderly individuals who live in their own home and have a comprehensive and complex need of care and support from different health care providers. The authors decided to interview in-patients at a geriatric clinic specializing in seniors with multimorbidity. Because of the complexity and heterogeneity in the health status of the seniors and their age-related pathologies, no single set of operational criteria was relevant to all research and clinical purposes (Valderas, Starfiled, Sibbald, Salisbury, & Roland, 2009). The authors used a narrower definition of multimorbidity and selected individuals with severe, documented illnesses who required informal and formal care from nursing aides and district nurses and had undergone intermittent acute hospital admissions. The operational definition of multimorbidity was: 75 years of age, hospitalized at least three times in the past 12 months, and meeting the criteria for three or more diagnoses (Gurner & Thorslund, 2003; National Board of Health and Welfare, 2002) based on the International Statistical Classification of Diseases (ICD-10). Other criteria were ability to speak Swedish and average hearing. Exclusion criteria were diagnosed dementia or acute confusion that could cause problems when the interviewer raised the subject of mental health. Data on diagnoses and hospitalization were collected from medical records. Seventy-three in-patients were asked if they would be willing to participate and 21 agreed, of whom five subsequently died and three declined when the interviewer contacted them. The final sample thus included 13 participants, who differed from each other in terms of age, sex, family structure, education, and diagnosis (Table I). They ranged in age from 79 to 96 years, lived in the community, while the two men resided with a partner and all the 11 women lived alone.

**Table I:** Characteristics of the study population (*N=*13).

Variables	*n*
Age (years)	85^a^ (79–96)
Sex	
Women	11
Men	2
Family structure	
Widowed and living alone	10
Divorced and living alone	1
Widowed and living with one child	1
Married	1
Education	
Secondary school	7
Upper secondary school	5
University	1
Diagnosis	
Heart failure	8
Hypertension	6
Hypothyreosis	6
Arrythmias	5
Asthma	5
Ischaemic diseases	5
Renal failure	3
Diabetes mellitus, type 2	3
Aortic stenosis	2
Mixed anxiety and depressive disorder	2
Sicca syndrome	2
Malignancy	1
Primary biliary cirrhosis	1

a Median values (range).

### Data collection

Semi-structured interviews were used to allow new viewpoints to freely emerge, thus achieving the aim of the study. The main two questions were: “How do you perceive the concept of mental health?” and “What do you consider might influence mental health?” Follow-up and probing questions were posed about the meaning of mental health, the participants’ own perceived mental health and what they regarded as important for the mental health of themselves and others. To strengthen the validity of the findings, the interviewer summarized the content after each interview and asked if the intended meaning had been captured. The interviews, which lasted from 30 to 50 min, took the form of a conversation and were conducted in the participants’ homes 1–3 months after discharge from the geriatric clinic. The interviews were audio-taped and transcribed verbatim before analysis. Data were collected from April to September, 2009.

### Analysis

The analysis of phenomenographic studies could be carried out in different ways. However, the structural and referential aspects of the studied phenomenon are essential. That is, we studied both the “what aspect” of the phenomenon and the “how aspect” of it. When the informants talk about this phenomenon: what do they talk about and how do they talk about it? The first author conducted the analysis and the other authors served as co-evaluators in the data categorization process. The data analysis in this study was conducted in seven steps in accordance with Dahlgren and Fallsberg (1991). The first step, *familiarization*, meant that the first author read the transcripts several times to obtain an overall impression and correct errors. In the second step, *condensation*, the first author tried to identify the most significant statements that gave answers to the two main interview questions. In these passages it was investigated *what* is in the focus of the informants attention and *how* she/he described the concept of mental health and what may influence it. The statements were then condensed in order to provide a short, but representative version of the entire dialogue about the concept of mental health and what may influence it. The concept of mental health is illustrated by the following excerpt from one of the informants:

I think mental health is a difficult concept … mental health … shall you be suffering … I also think that is a scary word – mental health! Because I think about mental problems and so … and I don't have that! … but maybe it becomes lonely and boring … maybe it becomes mental then …. (Informant 1)

The above cited interview mainly revealed that the concept of mental health was a difficult concept to describe (the “what” of the understanding) and that mental health was compared with mental health issues among other people who became lonely (the “how” of the understanding).

Mental health was also seen as a desire and could be improved upon, which is illustrated by following excerpt from one of the interviews:

Mental health is about … taking care of oneself and eating properly and … sleeping well and … not smoking and drinking booze or being careless … I have never done that! (Informant 2)

To illustrate another view of mental health and that it can be improved through being optimistic, an informant said:

Mental health? Yeah, that is when (laughing) a person seemed to be cheerful … are glad … taking everything in an easy way … (drinking water) … and thinking that everything is going to work out in a positive way! (Informant 4)

These two interviews revealed mental health being viewed as wanted behaviours or emotions (the “what” of the understanding) which may be achieved if a person takes care of oneself and had healthy behaviour or the ability to have positive thoughts (the “how” of the understanding).

The third step of the analysis involved *comparison* of significant statements in order to identify agreement or variation in the statements. In the fourth step, *grouping*, the authors (ÅG, BE, DR) grouped the conceptions into categories, based on similarities and differences, and then by formulating preliminary categories of description. The fifth step, *articulating*, represented an initial attempt to describe the essence of the similarities of understanding the phenomenon. In the next step, *labelling*, two authors (ÅG, MAD) labelled the categories so that the terms used provided a suitable description of the variations revealed by the informants. The final step, *contrasting*, involved all four authors comparing the categories in terms of differences and similarities, the purpose being to structure the outcome space and clarify the internal relationships between the categories and the structure of the variation in perceiving the concept of mental health and what may influence it.

### Ethical review

This study was approved by the regional ethics board (No. 2008/149-31). Both verbal and written information about the study was given to each in-patient invited to take part. They were also informed that participation was voluntary and could be terminated at any time.

## Findings

Six qualitatively different ways of understanding the concept of mental health and factors that may influence it reflecting key variations of meaning were identified. These different understandings form an outcome space of six descriptive categories that characterize the variation in conceptions of mental health (Table II). The commonalities and variations in the respondents descriptions are illustrated with excerpts from the transcripts. The quotes may provide illustrative examples of the category that supplements the abstract description. In a contrasting analysis, the structure of the variation in the outcome space is portrayed as the categories are compared, related to each other and discussed in relation to previous research. The discerned categories were as follows:

**Table II:** Contrastive analysis of the outcome space. Conceptions of mental health and dependent factors.

	Positive	Negative
Emotions	Dependent on desirable feelings and social contacts	Dependent on undesirable feelings and social isolation
Thoughts	Dependent on power of the mind and ability to control thoughts	Dependent on powerlessness of the mind and inability to control thoughts
Actions	Dependent on active behaviour and a healthy lifestyle	Dependent on passive behaviour and physical inactivity

### Mental health is dependent on desirable feelings and social contacts

The respondents explore desirable feelings related to the concept of mental health and the fact that social contact promotes it. Mental health was described as a sense of harmony, energy, strength, and feeling good. The respondents reported that these emotions enhanced their mental health. Emotions were considered to be relationship-oriented and mental health was dependent on a social network where relationships were based on trust. Most of the respondents reported that having somebody (e.g., children, friends) to talk to on a regular basis promoted mental health. It did not have to be face-to-face communication, as a telephone conversation was sufficient to prevent mental problems such as anxiety. Meetings with friends and relatives could improve mental health but it was essential that these meetings took place in the respondents’ home if such contact evoked undesirable feelings and thoughts such as worry about relatives or their illnesses:

I think that if I had someone to talk to I would feel a lot better …. If I only had an opportunity to talk to somebody about how I feel. (Informant 10)

### Mental health is dependent on undesirable feelings and social isolation

Emotions were described in terms of feeling bad or worried, which was regarded as poor mental health. Feelings were considered stress reactions resulting from personal strain or losses that negatively affected mental health. Most respondents denied feeling bad despite worrying a great deal about their relatives, financial situation and whether they could continue to live in their own home. One woman expressed a desire to share these feelings:

I feel worried when I'm lonely …. Nobody to talk to and you cannot call your children in the middle of the night and … and just ‘blabbing’ about (laughs) not feeling … yes … that you're feeling worried. (Informant 12)

Feeling down was another term used to describe the concept of mental health:

Well, mental health it's when you're feeling down (silence) …. I haven't been through that and so … but … but sure there is … in your surroundings …. Well, then you see everything as black. (Informant 2)

Emotions were also experienced as being associated with relationships as social isolation can lead to poorer mental health. Loneliness was another undesirable emotion. Being alone was described as having few or no contacts (e.g., close relatives of a similar age, spouse, friends) because most had died. The majority of the 13 informants lived alone (to which they were not accustomed) and felt lonely. This isolation led to worry about problems they had to solve on their own and they considered that their mental health would be better if they were less isolated: “Well, I have to say that one worries a lot more when one is alone ….” (Informant 12).

### Mental health is dependent on power of the mind and ability to control thoughts

The respondents also explore mental health as the power of the mind and ability to control thoughts, thus leading to better mental health. If a problem was considered unsolvable, it was necessary to contain any undesirable thoughts that surfaced. An optimistic view of life and a positive state of mind were important. Being optimistic was described as a personal trait and a positive state of mind enabled them to see life from a different perspective. They considered that they should not complain about unimportant matters but have positive thoughts and an affirmative attitude to life. The ability to find joy in life was based on a personal effort to maintain a cheerful outlook and to please or help other people. Joy in life was described as positive thoughts that could be developed by everybody, irrespective of age and sex. One way to achieve joy in life was to be surrounded by people with whom one could share positive thoughts or memories: “If I want good mental health… I have to please others” (Informant 4).

### Mental health is dependent on powerlessness of the mind and inability to control thoughts

Mental health was also described as powerlessness of the mind and an inability to control undesirable thoughts that were likely to lead to mental illness. The concept of mental health was associated with mental illness, which was conceived as something that people were born with or that developed through a process in the brain. Inability to control the mind was also reported to lead to negative thoughts about the future such as worry about one's financial situation, disease, and pain, which often happened when feeling lonely. The respondents used different strategies to rid themselves of negative thoughts (e.g., phoning someone), described as a consequence of poor mental health or inability to control the mind: “Yeah, mental health is actually … it's … about if you sort of can't control your own thoughts” (Informant 12).

In addition, the respondents described a fear of developing dementia, which meant a decreased capacity to think and remember things, in turn leading to mental illness such as depression. This idea was associated with experiences of meeting people with dementia as well as the impression that their own mental capacity had deteriorated compared with previous years. They also expressed a fear of not remembering details and names when talking to other people. Memory loss was particularly challenging for a person who already had poor mental health. Good mental health was described in terms of *sharpness* or *clearness*, which included the head: “I must say that my mental health means that I have a clear head …” (Informant 1).

Mental health was also explicitly associated with a neurological process in the brain: “I think that it mainly has to do with the nerves in the head” (Informant 9). This neurological process affected mental health in a negative way and mental illness was seen as the reason why people had lost their capacity to think or act in a normal way. Some informants reported feeling a *whirl* or *pressure* in their head and expected their mental health to deteriorate as a consequence. Mental health was also described in terms of mental illness, which was seen as a stigma:

I call them people with mental illness. That's my view …. And none of these people acknowledge that they have such problems. I don't agree. I suppose it's … in earlier years it was something shameful. (Informant 9)

### Mental health is dependent on active behaviour and a healthy lifestyle

The respondents also compared mental health as an active behaviour that leads to improved mental health. The concept of mental health was described in terms of *well* or *healthy*, which meant the absence of mental or physical disorders. A good physique and a healthy lifestyle were prerequisites for maintaining health and preventing disease. Active behaviour implied active choices and actions, which enabled an active and healthy lifestyle. It included the perception that good mental health required a healthy lifestyle with no drugs, tobacco, and only a moderate amount of alcohol. A person with good mental health was perceived as having good body function and physical health: “Mental health means that you are healthy and alert… and that you don't have any disorders” (Informant 2).

Physical activities were also described as important for enhancing a person's mental and physical health. Most of the respondents expressed that they wanted to do the things they had done before but were unable to do so because of lack of energy, fatigue, poor eyesight, decreased capacity to concentrate or reduced mobility. They wanted exercise (e.g., walking, dancing, swimming) but needed help from others because of functional disabilities. Some respondents stated that they no longer had any interests and one woman said that she was depressed and could not afford her former activities:

If I could just go somewhere to sit down and listen to a little music or something …. There is nothing I can do, I can't afford it …. (Informant 10)

A healthy lifestyle also meant a balance between physical activity and relaxation. Lack of sleep was perceived as a reason why some people had mental problems. One woman narrated that she had advised a neighbour who was crying and suffering from anxiety to go to sleep:

My neighbour got better after I told her to go to bed. She later came down to me and said: ‘you are the best doctor as I am much better today’ …. Maybe she was suffering from lack of sleep …. (Informant 11)

### Mental health is dependent on passive behaviour and physical inactivity

This conception illustrates that inability to engage in physical activity can lead to mental illness such as depression and dementia. Passive behaviour was difficult to change and weakened the ability to take action and responsibility for one's health. Mental health was also described in terms of being depressed or suffering from dementia, which was considered uncontrollable and dangerous in others. One participant asked herself:

What is mental health? …. Perhaps you can't control what you're doing … which makes you behave in a strange manner. (Informant 2)

Other respondents described their own experiences of being depressed and depression was seen as a mental problem inextricably linked to one's physical symptoms (e.g., tiredness and fatigue). This meant inactivity and feelings of hopelessness, which changed eating and sleeping patterns. It also implied difficulties finding a balance between physical activities and relaxation. Reduced mobility meant inability to continue various psychical activities. According to the respondents, mobility problems lead to a strong dependence on others and possibly also inability to maintain the home. A woman said:

Yes, for us who can't move in the same way as before, I think it's obvious that mobility is very important for mental health. (Informant 5).

Most of the respondents considered the term mental health to be scary or shameful and something that one could not discuss with others. The concept of mental health was also described as disorders such as Alzheimer's disease, about which most of the respondents were frightened of, as they did not want to become a burden on their family members. Feelings of depression and anxiety were often dominant, especially when considering a future replete with other diseases. The participants were of the opinion that physical conditions affected both physical and mental health in a negative way. The thought of developing other disorders made them fear severe bodily pain, leading to passive behaviour. Some participants expressed that their previous and present pain was something they expected with ageing itself and chronic pain was also the reason why some of the respondents felt depressed: “I'm thinking about how it affects you when you are in pain! It really affects you … mentally … as well” (Informant 12).

There were also respondents who stated that their mental health had deteriorated over the years, which they considered a normal part of ageing. A woman commented that she had heard that mental illness was common among elderly people and her physician had told her that depression was a natural part of an elderly person's life. Other participants argued that one could neither influence the ageing itself nor improve one's mental health: “No, that is not possible because mental problems appear when you get old ….” (Informant 11).

## Contrasting analysis of the outcome space

When we compare the six categories of the outcome space, we can note that a common characteristic of all categories are that they portray mental health as a relational concept, as dependent on different factors that could influence mental health in a positive as well as a negative sense. These factors can be described as emotions, thoughts and actions. The contrasting analysis is summarized in Table II. When mental health was characterized as desirable emotions, power of the mind, and active behaviour which were regarded as related aspects but when described as undesirable emotions; powerlessness of the mind and passive behaviour were associated. Desirable emotions prevented undesirable emotions, thoughts and actions that made it difficult to remain physically and socially active. Undesirable emotions were related to powerlessness over emotions, thoughts, and actions that led to poorer mental health. Mental health was also influenced by desirable and undesirable thoughts. Desirable thoughts meant the ability to control thoughts that would help a person to remain in good mental health. Undesirable thoughts meant inability to control thoughts, thus leading to poorer mental health. Mental health was also characterized as a behaviour that may affect a persons health in a positive or a negative way. An active behaviour meant the ability to control one's health and live a physically active life that would promote it. A passive behaviour meant inability to control health and living a physically inactive life that would result in a poorer mental health.

## Reflections

### The concept of mental health

The most significant finding was that mental health was mainly perceived in terms of mental health issues or illnesses such as depression and dementia. Elderly people tend to describe mental health in a negative way or in relation to societal norms and regulations (Hedelin & Strandmark, 2001a). When the participants mentioned depression it was often connected to their own symptoms of depression and the fear of feeling or being depressed. These informants’ conception of mental health, or second-order perspective, should be viewed in light of the fact that some participants had been diagnosed with mixed anxiety and depressive disorder according to the informants’ medical journals, that is, the first-order perspective (Marton & Booth, 1997). There were also participants who reported that they had experience of depression among others and that poor mental health was one reason why other people had impaired cognition. Mental health was also compared with dementia, which was described as disturbances in brain function. They seemed particularly concerned about memory loss and cognitive impairment, indicating the connection between depression, medical problems, and cognitive impairment (Samuelsson et al., 2005). The respondents viewed mental health as desirable or undesirable emotional experiences. To our knowledge, no other studies have shown that mental health among elderly people presents these opposite emotional experiences. However, mental health was not only described in negative terms, demonstrating that the border between mental health and mental ill health might be difficult to define (Hedelin & Svensson, 1999). Mental health was also mentioned in positive terms, which could be similar to a description by Hedelin and Strandmark (2001b). Mental health was further described as being healthy without any physical or mental disease. Some participants had a more physical and holistic view on mental health which indicates a perceived interrelation between mental and physical health. Mental health can also be compared to the domains of HRQOL. The concept of HRQOL provides a multidimensional perspective encompassing an individual's physical, emotional and social functioning (McDowell & Newell, 1996).

### Factors that may influence mental health

The participants mentioned perceptions that had either a negative or a positive impact on mental health. The most frequent were that social contacts can improve and that social isolation might worsen it. The majority of the participants lived alone, experienced loneliness and expressed that they missed former social contacts. There is evidence that older women with lower emotional and instrumental support are more likely to be depressed compared with a similar group of men (Gautam, Saito, Houde, & Kai, 2010). Loneliness is a common source of suffering in older persons and it has recently been demonstrated that loneliness *per se* is a predictor of functional decline and death (Perissinotto, Stijacic Cenzer, & Covinsky, 2012). The present findings reveal that some participants claimed to be unable to take part in social and physical activities because of disabilities and other limitations. They also believed that being able to engage in their favourite activities would have a positive effect on their own mental health. These findings support the fact that physical and social activities can improve elderly individuals’ social network, as well as their psychological and physical well-being (Greaves & Farbus, 2006). The inability to maintain activities is therefore important for detecting depression among older persons (Martin et al., 2008). Physical activity is strongly associated with improved mental health among seniors who reside in their own home (Lautenschlager, Almeida, Flicker, & Janca, 2004). Being physically active in later life is also strongly associated with a lower risk of depressive symptoms, which has implications for mental health promotion in aged populations (Ku, Fox, Chen, & Chou, 2012). The participants in this study expressed a fear of dementia, and physical activity may reduce the risk of or delay dementia, although it cannot be prevented by means of physical activity (Benedetti, Borges, Petroski, & Goncalves, 2008). These findings are in line with several studies that found a significant relationship between the level of physical activity and mental health status (Benedetti et al., 2008) and that free interventions focusing on social support should be considered in mental health promotion among older adults living at home (Bøen, Dalgard, & Bjertness, 2012).

Despite this knowledge, there is a lack of a structured approach when district nurses in primary care promote physical activity among senior citizens who live in the community (Goodman, Davies, Dinan, See Tai, & Iliffe, 2011).

Some participants in this study reported that positive thinking and being optimistic about life enhanced mental health. Greaves and Farbus (2006) found that promoting social activity and optimism about life among the elderly led to positive changes in health behaviour. Being optimistic can have a positive effect on mental and physical health, as well as facilitating coping with everyday life (Conversano et al., 2010). Other researchers have demonstrated that positive thinking is the most common process for promoting one's own health (Björklund, Sarvimäki, & Berg A, 2008). However, some participants in our study expressed the view that ageing itself has a negative effect on mental health. One explanation could be their expectations about “old people”. Getting older is often associated with physical deterioration and depression, which are regarded as a “normal” part of ageing (Conner et al., 2010). Depression among seniors with long-term physical conditions may be regarded as a “natural” reaction to a patient's life circumstances even when it is identified (Unützer et al., 2000). A previous study showed that having a higher expectation about ageing was associated with better mental and physical health, after adjusting for gender, age, and education (Kim, 2009). Some of the respondents in the present study expressed that they were suffering of chronic pain and they seemed to think that this pain was a natural element of disease and ageing itself. One respondent also expressed that pain was the reason why she felt depressed. Nevertheless, chronic pain and mood disorders are common in older people. A previous study revealed that painful physical symptoms were strongly and independently associated with a 12-month major depressive episode and that the presence of pain influenced help seeking behaviour and use of psychotropic medication (Bonnewyn et al., 2009). In addition, primary care providers should recognize that pain is a common symptom of depression. Moreover, painful conditions and depression frequently coexist and evaluation and treatment of both are important (Bair et al., 2003).

Our findings indicated that both social and physical activities are a cornerstone of good mental health and that positive thinking is crucial for promoting mental health. Kim (2009) argued that nurses need to implement interventions that promote expectations of ageing and health-promoting behaviours for good mental and physical health in later life. For instance, there are social and psychological models which assume that positive illusions promote good mental health (Gana et al., 2004). There is also an increasing interest in improving health by changing patient behaviour through self-management support (Wagner & Groves, 2002). Self-management may be described as a process in which patients learn and then use skills to improve their physical and emotional well-being, despite their disease. Self-management includes the patient as partner in his or her own care (Dickerson et al., 2011). Nevertheless, it is also essential to establish guiding public health policy actions in order to promote and maintain active and healthy ageing with a better quality of life (Benedetti et al., 2008). When it comes to community-oriented work and interventions directed towards ill health which are related to perceptions and emotions, there is a mighty need for knowledge and developments of different methods (Hedelin & Svensson, 1999). Supplementary reinforcement involving contact by health professionals may be a prerequisite to the effectiveness of IT-based delivery systems for health promotion in seniors (Harari et al., 2008). However, for promotion of physical activity by seniors to be meaningfully incorporated into primary care nursing work, there is a need to develop a more strategic approach. The focus would be on optimizing the opportunities and interest of primary care nurses and developing the knowledge and skills of the workforce in this area of nursing work (Goodman et al., 2011).

Loneliness is also a common well-known risk factor associated with mental health problems. Therefore, asking older persons about loneliness can also be a useful way of identifying seniors at risk of disability and poor health outcomes (Perissinotto et al., 2012) such as mental illness. The overall perceptions in the present study might be associated with a lack of power and control or how multiple chronic conditions affects the seniors’ own life situation and frequent hospitalizations. Seniors with multimorbidity may suffer from acute illness and they are therefore often hospitalized. The present study does not support that a high level of nervousness and lack of well-being among hospitalized older patients results from acute illness and hospitalization. The worsening in the score of well-being after discharge of such patients is primarily caused by low levels of feelings of security. This represents a challenge to nurses providing home-based care. Nurses, in hospital as well as in primary health care, may contribute to the identification of patients with issues such as anxiety and reduced well-being (Kvaal & Laake, 2003) when health promotion encourages well-being and thus is of positive value (Gorin & Arnold, 2006).

Finally, health promotion has been highlighted as an important task for the health care sector in Sweden, and district nurses are specially trained for and interested in this issue. However, these enrolled nurses may find it difficult to prioritize between disease-oriented and health promotion work, and this results in the time district nurses spend on health promotion being limited (Wilhelmsson & Lindberg, 2009). One other solution is implementing public health nurses in primary health practice. These nurses may foster citizen participation by acting on the determinants of health and implementing strategies to achieve longer-term health outcomes. Here, the relational work between clients and public health nurses created empowering processes whereby vulnerable citizens were supported to participate in assessing, planning, implementing, and evaluating their health and health care (Aston & Meagher-Stewart, 2009). The present study is in accordance with Hansson (2004), who defined empowerment as a process of enhancing people's abilities to meet their own needs, solve their own problems, and mobilize the necessary resources to take control over their life. Björklund et al. (2008) argued that empowerment is the goal of health promotion and both the individuals’ and the health professionals’ knowledge should be employed to identify strategies for each person's empowerment process.

### Methodological considerations

In order to obtain a varied sample, the study employed purposeful sampling. As the number of participants was low, it is likely that diversity of experiences was not captured by our operational definition of multimorbidity. The goals were to capture the perspective of patients with complex and overlapping health problems, thus it was important to take account of the severity and number of diseases, together with acute and intermittent hospitalization. Recruitment was difficult, and most of the potential informants declined because of fatigue or unwillingness to discuss mental health. In-depth interviews were appropriate as most of the informants found it difficult to talk about the subject of mental health, which they initially described as abstract and hard to understand. The reasonableness of the result was increased by the fact that two pilot interviews were conducted (Svensson, 1997). Although some of the interviews were short, the informants’ statements answered the two main questions. In view of the above-mentioned limitations, it can be questioned whether or not the concept of mental health is clearly defined in relation to what these elderly persons perceived as having a possible influence on mental health. In other words, the transferability of the results is limited because of the small and selective sample. Sjöström and Dahlgren (2002) noted that credibility concerns the relationship between empirical data and descriptive categories, i.e., the researcher has to clearly demonstrate that the description of differences and similarities is supported by the empirical material. This can be achieved by presenting the links between the findings together with each conception and its descriptive categories in a table with an outcome space. In phenomenographic studies, the outcome spaces are often hierarchically structured, where the conceptions reveal more or less complex understandings in relation to what is held as a “correct” answer. In this study, the outcome space can be described as horizontal and dichotomous, as the categories discern different aspects of mental health (i.e., emotions, thoughts, and actions), with a positive and a corresponding negative conception. We choose to provide short quotations from the interviews to illustrate the relevance of the categories. One question is whether the same categories would emerge if other researchers analysed our material. Marton (1988) has argued that the demand for replicability is not justified, or even desirable, as the actual identification and description of the categories constitute the “discovery” of the study (Sjöström & Dahlgren, 2002).

## Implications

Our findings highlight the importance of individual definitions of mental health and also that community-dwelling seniors with multimorbidity can describe how multiple chronic conditions affect their life situation. According to the respondents view, the concept of mental health can be defined as how an individual feels, thinks, and acts and also includes a positive as well as a negative aspect. It also reveals that social contacts, physical activity, and optimism may improve mental health while social isolation, ageing, and chronic pain may worsen it – according to the respondents. The most significant findings in the present study were that there were respondents who described mental health in negative terms and that mental health was perceived as something that one could not discuss with others. Other respondents perceived their own experiences of mental illness such as depression as “normal” and a “natural” part of the ageing process. These assumptions of depression in older age have to be corrected when the effective psychological and medical treatments are available (McEvoy & Barnes, 2007).

Detecting mental illness among seniors has become a challenge for both health professionals and the public sector. Prevention of late-life depression requires identification of seniors at highest risk, such as those with specific chronic medical conditions (Lyness, Yu, Tang, Tu, & Conwell, 2009), painful physical symptoms (Bonnewyn et al., 2009), and perceived loneliness (Perissinotto et al., 2012). Harrison et al. (2012) suggest that depression screening combined with a count of conditions would provide a useful assessment. After diagnosis comes the possibility for treatment and care. Bigger improvements in the quality of care may be achieved if services foster links with community resources and develop more efficient delivery systems via service redesign (McEvoy & Barnes, 2007). Engaging seniors in creative activities, using an individualized, mentor-based approach may be one way of improving social networks, re-connecting people with their local communities, and ultimately improving their physical and psychological well-being (Greaves & Farbus, 2006). Nevertheless, we have to remember that community-dwelling seniors with multimorbidity are a heterogeneous group. Therefore, the care for patients with multiple chronic conditions had to be patient centred and individualized which supports their unique collection of problems, shifting priorities, and multidimensional decision making (Bayliss et al., 2008).

The findings in the present study clearly present challenges to the organizations that provide care and services to community-dwelling seniors with multimorbidity. In Sweden, elderly people with multimorbidity often remain in their own home and are cared for by nursing aides and district nurses. Being able to stay in their own home as long as possible is the general preference for many elderly even those that live alone (Dale, Söderhamn, & Söderhamn, 2012). On the other hand, caring for seniors with mental disorders constitutes a complex situation in which distancing serves as a resource to protect oneself and to reduce the burden of caring (Martinsson, Wiklund-Gustin, Lindholm, & Fagerberg, 2011). Nevertheless, health promotion is an important task for the health care sector and district nurses are especially trained and interested in this issue. However, district nurses may be stuck between disease-oriented and health promotion work (Wilhelmsson & Lindberg, 2009). This implies that enrolled nurses such as district nurses need time, resources, and knowledge to identify risk factors as well as to improve mental health. A challenge ahead is supporting seniors to retain their optimism, as well as making social and physical activities more attractive and accessible to these individuals by taking account of their age, abilities, and interests. It is therefore essential to organize the health care system to provide individual health promotion dialogues for community-dwelling seniors with multimorbidity. Future research should address the prerequisites for conducting such mental health promotion dialogues among the community-dwelling seniors with multimorbidity.
